# Research on the prediction of blasting fragmentation in open-pit coal mines based on KPCA-BAS-BP

**DOI:** 10.1038/s41598-024-67139-x

**Published:** 2024-10-14

**Authors:** Shuang Liu, Enxiang Qu, Chun LV, Xueyuan Zhang

**Affiliations:** School of Architecture and Civil Engineering, Qiqihar University, Qiqihar, 161006 Heilongjiang People’s Republic of China

**Keywords:** Open-pit coal mine, Blasting fragmentation, Kernel principal component analysis (KPCA), Beetle antennae search algorithm (BAS), BP neural network, Engineering, Mathematics and computing

## Abstract

The blasting block size of open-pit mines is influenced by many factors, and the influencing factors have a very complex nonlinear relationship. Traditional empirical formulas and a single neural network model cannot meet the requirements of modern blasting safety. To improve the prediction accuracy of blasting block size, the measured data of Beskuduk open-pit coal mine is used as training and testing samples. Seven factors including rock tensile strength, rock compressive strength, and blast hole spacing are selected as input variables of the prediction model. The average size of blasting fragmentation X50 is used as the output variable of the prediction model. The kernel principal component analysis (KPCA) is adopted to reduce the dimensionality of the input variables. The beetle antennae search algorithm (BAS) is selected to optimize the parameters of the initial weights and thresholds of the back propagation (BP) neural network. Finally, prediction model of blasting fragmentation in open-pit coal mine based on KPCA-BAS-BP is established. The results show that the average relative error of the model is 1.77%, and the root mean square error is 1.52%. Compared with the unoptimized BP neural network and the BP neural network optimized by the artificial bee colony algorithm (ABC) model, this model has higher prediction accuracy and is more suitable for predicting the blasting block size of open-pit coal mines, it provides a new method for predicting the fragmentation of blasting under the influence of multiple factors, filling the gap in related theoretical research, and has certain practical application value.

## Introduction

Blasting fragmentation is an important indicator for measuring the quality of blasting. Reasonable blasting fragmentation is vital to improving the subsequent production efficiency of a mine and reducing the total production cost, it has always been a closely watched issue by many scholars^1^. The control of blasting rock fragmentation has always been a technical problem in the production process of open-pit mines. Given that blasting fragmentation is affected by many complex factors, describing them with precise mathematical formulas is difficult. In actual mine production, designing blasting parameters is often based on experience, so the blasting effect difficultly meets production needs^2^.

Artificial intelligence technology is constantly developing and applied in mining, and traditional empirical formulas and a single neural network model cannot meet the requirements of modern blasting safety^3,4^. At present, the main methods used by domestic scholars to predict blasting fragmentation include random forest regression^5^, extreme learning theory^6^, least squares support vector machine regression^7^, statistical classification discrimination^8^, and BP neural network^9^. Xia Shu yuan used out of bag estimation and mutual information (MI) methods from Random Forest (RF) for feature selection, and proposed an Extreme Gradient Boosting Tree (XG Boost) blast block size prediction model based on feature engineering^10^; Fan Yong analyzed the geometric characteristics of blasting funnels and the size of rock fragmentation under different burial depths and quality conditions of explosives, and used the Continuous Discontinuous Element Method (CDEM) to simulate the shape of blasting funnels and the size of rock fragmentation^11^; Based on the basic principle of BP neural network, Liu Ying ji uses PSO algorithm to optimize network weights and biases, constructs PSO-BPNN model, trains and tests the model with typical blasting data, and verifies the reliability and applicability of the model based on the actual engineering of Shanxi Hun yuan Pumped Storage Power Station^12^. BP neural network, with its excellent self-learning ability and generalization ability, is widely used by scholars in blasting block size prediction.

Aiming at the drawbacks of BP neural network, such as easily falling into local minima and slow training speed, this paper proposes to establish a blasting block size prediction model based on Kernel Principal Component Analysis (KPCA) and Beetle Antennae Search Algorithm (BAS) to optimize the BP neural network. Firstly, KPCA is used to reduce the dimensionality of the sample data, extract the comprehensive principal components that affect the blasting block size as input variables for the model, optimize the initial weights and thresholds of the BP neural network using the BAS algorithm, and finally establish the KPCA-BAS-BP prediction model. At the same time, it is compared with the unoptimized BP neural network and the BP model optimized by the Artificial Bee Colony Algorithm (ABC), and good prediction results are achieved.

## Case study

**Figure 1:**
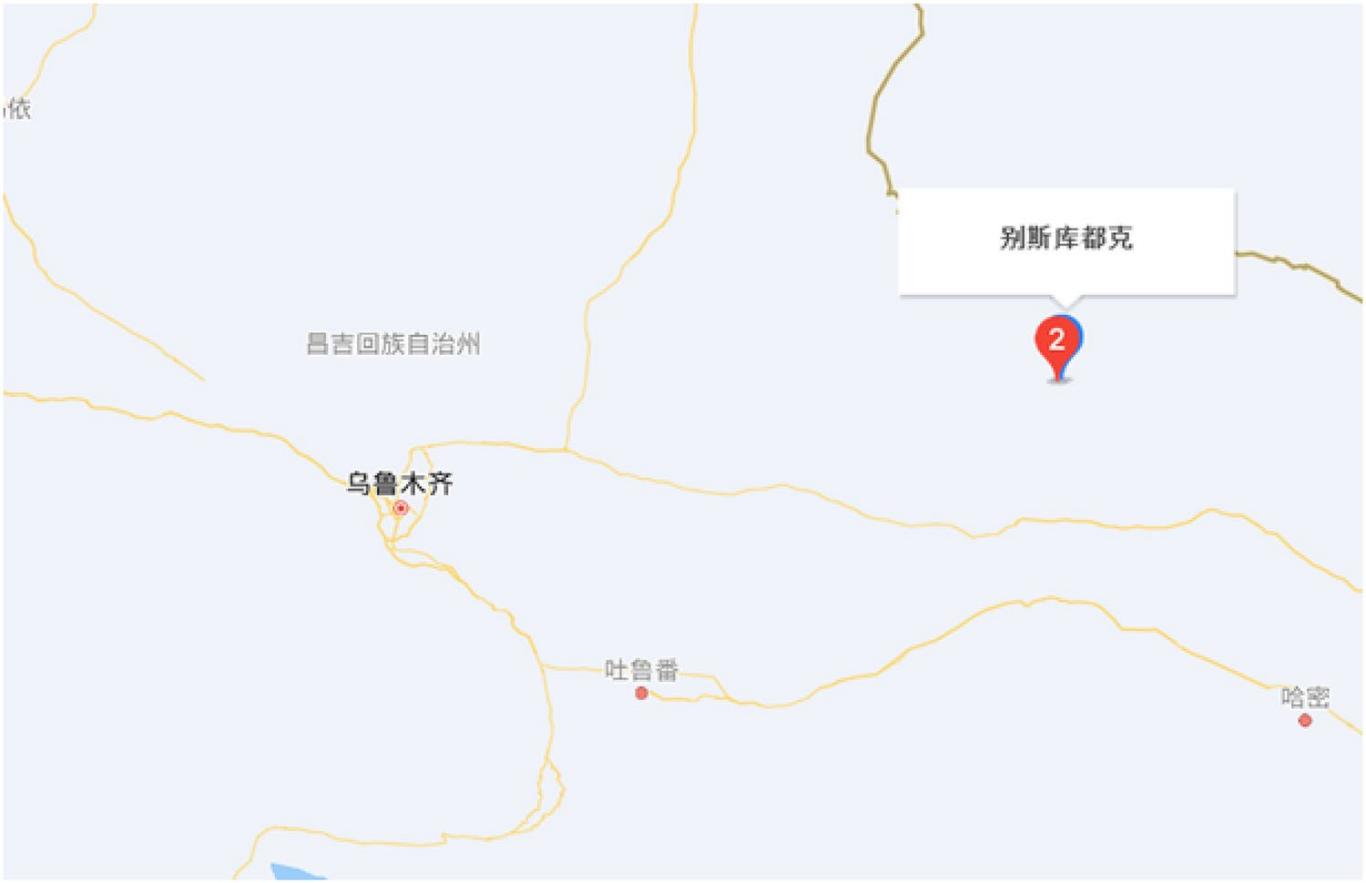
Geographical location of coal mines.

**Figure 2:**
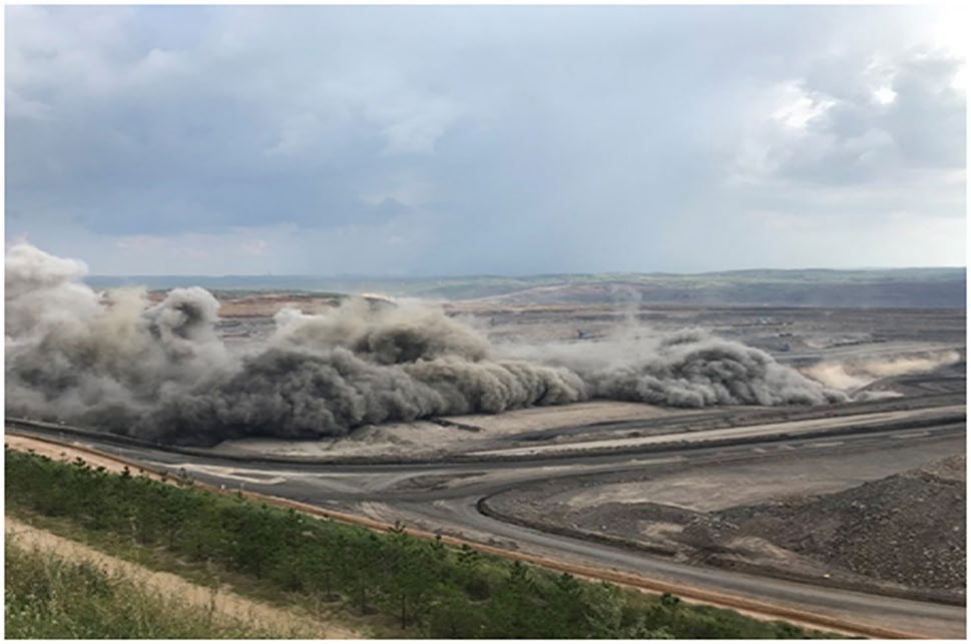
Blasting site.

**Figure 3:**
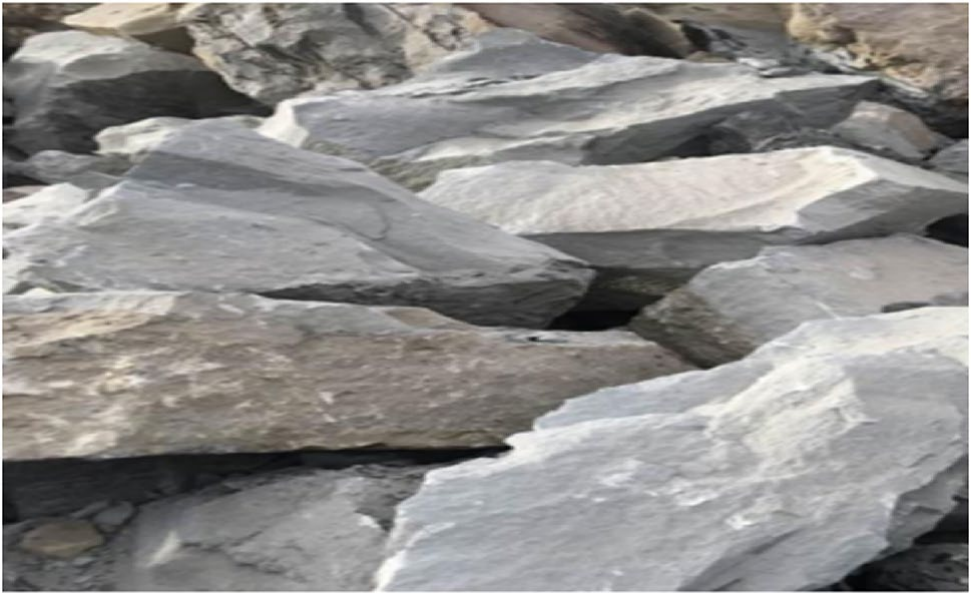
Blasting block pile.

The Beskuduk Coal Mine is located in Dahongliuxia Township, Balikun Kazak Autonomous County, Xinjiang Uygur Autonomous Region. It covers an area of 15.5 square kilometers, with a proven reserve of approximately 250 million tons and a designed production capacity of 3 million tons per year. The geographical coordinates of the mining area are: longitude 91°52′15″ to 91°55′30″ east and latitude 44°24′15″ to 44°26′45″ north (Fig. 1). The mining area is defined by 8 inflection points, with a mining elevation range of + 1310 m to + 800 m. The mining area is 0–4.5 km long from northwest to southeast, 0.7 km to 4.5 km long from northeast to southwest, and covers an area of 12.14 km^2^. The blasting site and pile of blasting blocks currently being mined in the mining area are shown in Figs. 2 and 3.

## Basic principle of KPCA-BAS-BP

### KPCA

Principal component analysis (PCA) uses the characteristic covariance matrix to evaluate the consistency of variance among variables. The optimum linear combination among variables is identified to replace the characteristics and thus achieve dimensionality reduction. However, this method involves linear-dimensionality reduction. If the relationship among features is nonlinear, it would be inefficient for describing them with a linear relationship. KPCA uses the idea of a kernel to map the sample space to a higher-dimensional space and then uses the higher-dimensional space for linear-dimensionality reduction.

Suppose ${x}_{i}\in {R}^{m}\left(i=\text{1,2},\dots ,n\right)$ represents an m-dimensional column vector, $\varphi$ is a nonlinear mapping function, and ${R}^{m}$ is mapped to the feature space $F$, then^13^

1 $$\varphi :{R}^{m}\to F,x\to \varphi \left(x\right)$$

The covariance matrix of the sample data in the feature space F is

2 $${C}^{F}=\frac{1}{n}\sum_{j=1}^{n}\varphi \left({x}_{j}\right){\varphi \left({x}_{j}\right)}^{T}$$

The characteristic equation of the simplified covariance matrix can be expressed as

3 $${C}^{F}v=\lambda v$$

where $\lambda$ and $v$ are the eigenvalues and eigenvectors of the covariance matrix, respectively.

The feature vector $v$ can be expressed as

4 $$v=\sum_{i=1}^{n}{\alpha }_{i}\varphi \left({x}_{i}\right)$$

The mapping vector $\varphi \left({x}_{k}\right)$ on both sides of Eq. (3) is multiplied at the same time, and the inner product operation is performed:

5 $$\lambda \left(\varphi \left({x}_{k}\right)\cdot v\right)=\varphi \left({x}_{k}\right)\cdot \left({C}^{F}v\right)$$

The kernel matrix is calculated as follows:

6 $${K=[K}_{ij}]=K\left({x}_{i},{x}_{j}\right)=\varphi {\left({x}_{i}\right)}^{T}\varphi ({x}_{j})$$

7 $$n\lambda \alpha =K\alpha$$

where $n\lambda$ is the eigenvalues of the kernel matrix $K$, and $\alpha$ is the eigenvectors corresponding to the eigenvalues.

The projection of vector $\varphi \left(x\right)$ on the feature vector $v$ is

8 $$\left(\varphi \left(x\right)\cdot v\right)=\sum_{i=1}^{n}{\alpha }_{i}\varphi {\left({x}_{i}\right)}^{T}\varphi \left(x\right)$$

The projection of the vector $\varphi \left(x\right)$ in the feature space $F$ is

9 $$\left(\varphi \left(x\right)\cdot v\right)=\sum_{j=1}^{n}{\alpha }_{i}K\left({x}_{i},x\right)$$

Principal components with a cumulative contribution rate of over 85% is used^8^:

10 $$\frac{\sum_{k=1}^{s}{\lambda }_{k}}{\sum_{k=1}^{m}{\lambda }_{k}}\ge 85\%$$

where $s$ is the number of pivots that meet the conditions.

The Gaussian radial basis kernel function $K\left(x,y\right)$ is selected:

11 $$K\left(x,y\right)={e}^{-\frac{{\left|\left|x-y\right|\right|}^{2}}{{2\sigma }^{2}}}$$

### BAS

BAS^14^ algorithm is a algorithm first proposed in 2017 to achieve global optimization of multi-objective functions based on beetle foraging. The basic principle is that when an individual is looking for food, it does not know the specific location of the food. Instead, the individual searches for the target according to the smell of the food. The beetle has two long antennae on the left and right. During the foraging process, if the intensity of the smell received by the left antennae is greater than that received by the right, the beetle would fly to the left; otherwise, it will fly to the right^15^. Similar to genetic algorithm and particle swarm optimization algorithm, BAS can automatically realize the optimization process without knowing the specific form of the function and gradient information. Moreover, only one individual exists, and the optimization speed can be significantly improved. The optimization steps are as follows.

Step 1: A random vector of the direction of the left and right antennae of the beetle is established and normalized.
  12 $$dir=\frac{rands(k,1)}{\parallel rands(k,1)\parallel }$$
  where $rands()$ is a random function, and $k$ is the space dimension.
Step 2: The spatial coordinates of the left and right antennae of the beetle are created.
  13 $$\left\{\begin{array}{c}{x}_{r}={x}^{t}+{d}_{0}\frac{dir}{2}\\ {x}_{l}={x}^{t}-{d}_{0}\frac{dir}{2}\end{array}\right. (t=\text{0,1},2\cdots ,n)$$
  where ${x}_{r}$ is the position coordinates in the search area of the right antennae, ${x}_{l}$ is the position coordinates in the search area of the left antennae, ${x}^{t}$ is the center of mass coordinates, and ${d}_{0}$ is the distance between two antennae.
Step 3: According to the fitness function $f\left({x}_{r}\right)$ and $f({x}_{l})$, the strength of the smell to the left and right antennae of the beetle is evaluated.
Step 4: The position of the beetle according to the evaluation result is iteratively updated.
  14 $${x}^{t+1}={x}^{t}-{\delta }^{t}*dir*sign(f\left({x}_{r}\right)-f({x}_{l}))$$
  where ${\delta }^{t}$ is the step factor at the tth iteration, and $sign ()$ is a symbolic function.

### BP neural network

BP neural network is a feedforward neural network that uses BP algorithm to dynamically adjust weights and thresholds during learning and training. This algorithm has good self-organization, self-adaptation, and nonlinear mapping capabilities. It can also process complex linear and nonlinear relationship data and predict various targets requiring estimation^16^. The topological structure of a BP neural network generally includes the following: input layer, hidden layer(s), and output layer. The network structure can be adjusted by increasing the number of hidden layer units. The basic algorithm includes the forward propagation of the signal and the BP of the error. The gradient descent method is used to dynamically adjust the network weights and thresholds to minimize the mean-square error (MSE) between the network output value and the expected one. Learning and training are repeated until the preset number of learning iterations is reached or the preset training output error is met^17^.

## KPCA-BAS-BP predictive model

**Figure 4:**
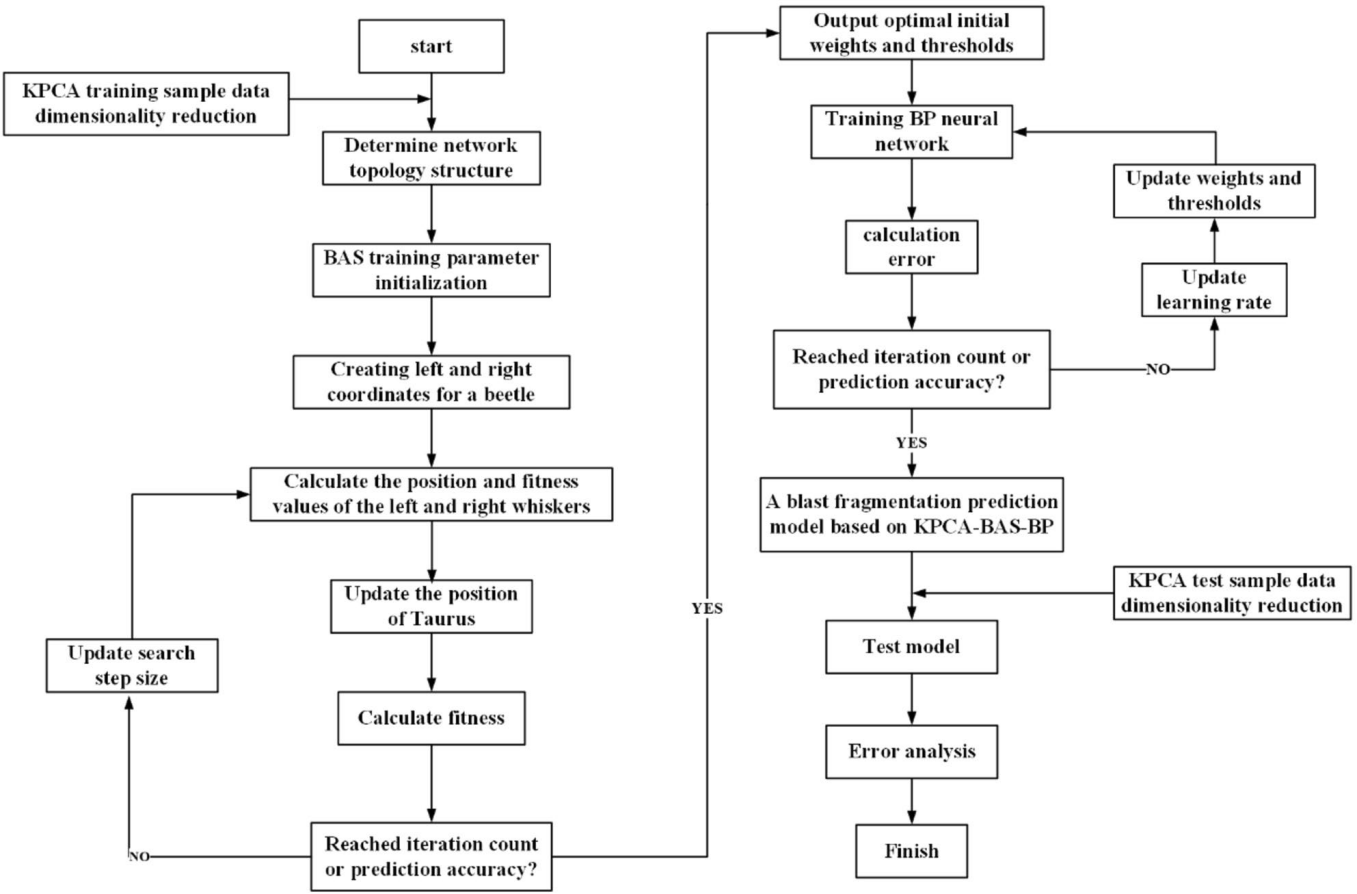
Flow chart of KPCA-BAS-BP model implementation.

KPCA is used to reduce the dimensionality of the sample data, and the BAS algorithm is used to optimize the initial weights and thresholds of the BP neural network. Then, the BP neural network is used to establish the nonlinear relationship between the impact factors and the blasting fragmentation. Ultimately, the model is formed based on the KPCA-BAS-BP prediction model of blasting fragmentation in an open-pit coal mine. The specific flow chart is shown in Fig. 4.

The steps based on the KPCA-BAS-BP model are as follows.

Step 1: KPCA is used to reduce the dimensionality of training sample data.
Step 2 : A random vector of the direction of the beetle antennae is established, and the search space dimension is defined. The structure of this model is 5 input layer neurons, 10 hidden layer neurons, and 1 output layer neuron. The spatial dimension $k=5*10+10*1+10+1=71$.
Step 3: The step factor $\delta$ is set. In the iterative process, the variable step length is calculated by formula (15), where $eta$ is generally 0.95.
  15 $${\delta }^{t+1}={\delta }^{t}*eta t=(\text{0,1},2,\dots ,n)$$
Step 4: The fitness function is determined. In the KPCA-BAS-BP prediction model, the MSE of the training data is used as the fitness-evaluation function to realize the search of the spatial region.
  16 $$fitness=MSE=\frac{1}{n}\sum_{i=1}^{n}{(y-{y}_{i})}^{2}$$
  where $n$ is the number of input samples, $y$ is the expected output value, and ${y}_{i}$ is the actual value.
Step 5: The beetle position is initialized. The initial parameter $X= rands(k,1)$ is selected as the initial position of the beetle and saved in $bestX$.
Step 6: The fitness-function value of the initial position of the beetle is calculated and saved in $bestY$.
Step 7: The positions of the left and right antennae and the optimal solution are updated. According to the positions of the two antennae, the fitness-function values $f({x}_{l})$ and $f({x}_{r})$ of the left and right antennae are solved, and their values are compared to update the beetle position. The fitness-function value is calculated at this time, and the fitness-function value is updated if it is better than $bestY$^18^.
Step 8: Whether the fitness-function value meets the preset iteration accuracy or whether it reaches the maximum number of iterations is determined. If met, the next step is performed, otherwise; step 7 is repeated to continue the iteration.
Step 9: The optimal solution is outputted. The obtained optimal initial weights and thresholds are brought into the BP neural network for training, and the prediction model is built.
Step 10 : KPCA is used to reduce the dimensionality of the test sample data, which are then inputted into the established prediction model to obtain the prediction result and perform error analysis.

## KPCA-BAS-BP prediction-model application and analysis

### Sample collection and selection of model-evaluation indicators

This article selects 60 sets of measured data from different blocks of the Beskuduk open-pit coal mine for shuffling processing. The rock tensile strength ($MPa$), rock compressive strength $(MPa)$, blast-hole spacing ($m$), row spacing ($m$), minimum resistance line ($m$), ultradepth ($m$), and explosive unit consumption (kg/m^3^) are selected as the input variables of the prediction model. The average size of blasting fragmentation X50 ($m$) is selected as the output variable of the prediction model^19^. The standardization of the 60 sets of sample data is shown in Table 1.

**Table 1:** Standardization of data of blasting fragmentation.

	X_1_	X_2_	X_3_	X_4_	X_5_	X_6_	X_7_	Y
1	− 0.10941	− 2.34687	− 0.59235	0.57252	0.24007	− 1.09349	− 0.01274	− 0.96246
2	0.15854	− 2.27124	− 0.59235	0.57252	0.24007	− 1.09349	− 0.01274	− 0.66733
3	0.82842	− 2.01911	− 0.59235	0.57252	0.24007	− 1.09349	− 0.01274	− 0.27382
4	− 0.64532	− 1.51486	0.19745	0.57252	1.08736	− 1.09349	1.26120	1.00509
5	− 1.71713	− 1.51486	1.51379	0.57252	− 1.17209	− 0.23585	0.75163	− 1.94624
6	− 0.51134	− 1.48965	0.19745	0.57252	1.08736	− 1.09349	1.26120	1.10347
7	− 1.71713	− 1.31316	1.51379	0.57252	− 1.17209	− 0.23585	0.75163	− 1.84786
8	− 0.10941	− 1.31316	1.51379	0.57252	− 1.17209	− 0.23585	0.75163	− 0.37220
9	− 1.44917	− 1.01061	1.51379	0.57252	− 0.32480	1.05061	− 1.28668	− 0.86408
10	− 0.64532	− 0.85933	0.19745	0.57252	1.08736	− 1.09349	1.26120	1.20185
11	− 1.3152	− 0.83412	− 1.11889	− 1.71756	1.36979	1.05061	0.24205	0.11969
12	− 0.51134	− 0.8089	− 1.11889	− 1.71756	1.65222	− 0.23585	1.00641	1.00509
13	− 1.44917	− 0.75848	1.51379	0.57252	− 0.3248	1.05061	− 1.28668	− 0.76571
14	− 0.24339	− 0.73327	− 1.11889	− 1.71756	1.65222	1.05061	− 0.01274	1.30023
15	0.69444	− 0.73327	0.19745	0.57252	− 0.04236	1.05061	− 0.01274	− 0.27382
$\ldots$	$\ldots$	$\ldots$	$\ldots$	$\ldots$	$\ldots$	$\ldots$	$\ldots$	$\ldots$
60	1.13642	1.71236	− 1.11889	− 1.71756	− 0.60732	− 1.09349	− 1.79626	0.61158

### KPCA-BAS-BP prediction-model establishment

**Figure 5:**
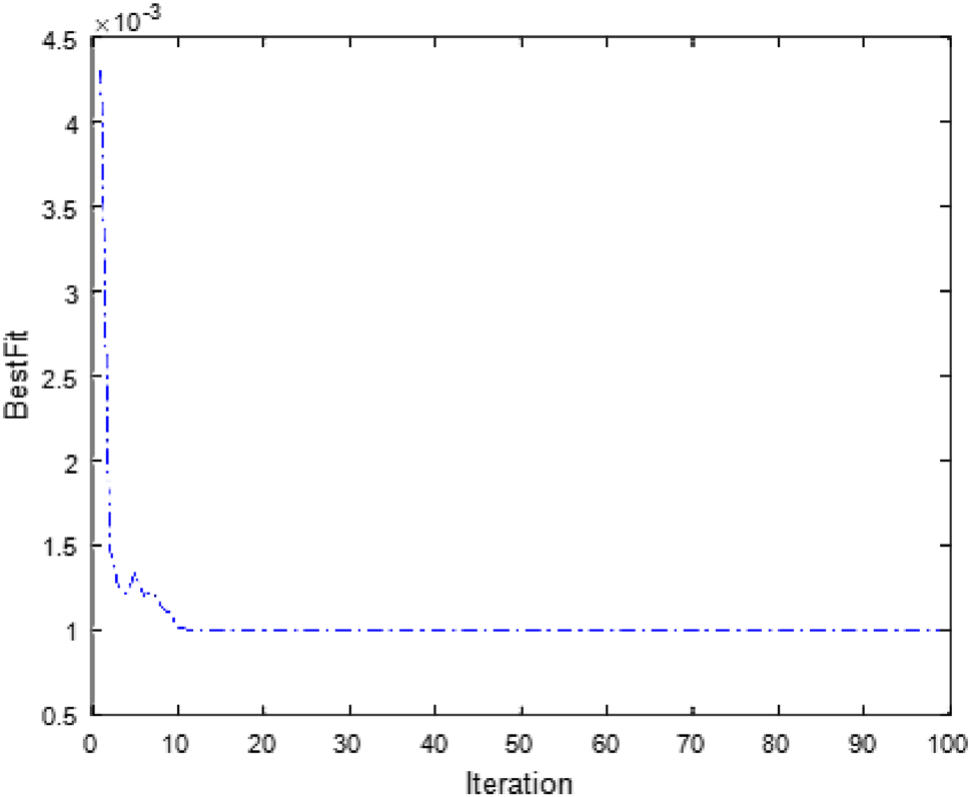
Fitness curve of KPCA-BAS-BP model.

Matlab2016 (a) is used to establish the prediction model. The parameters of the KPCA-BAS-BP prediction model are set as follows. The Gaussian radial basis function is used as the kernel function of KPCA to reduce dimensionality. The initial step length of the BAS algorithm (*step*) is 1, and the relationship between the step length and the initial distance (*c*) is 5. The number of iterations (*n*) is 100. Moreover, the number of the input layer neurons of BP neural network is 5, the number of the hidden layer neuron is 10, the number of the output layer neuron is 1, the maximum training iterations is 1000, the learning rate is 0.01, the training accuracy is 0.001, and the momentum coefficient is 0.9. The first 45 sets of data are selected as learning samples to train the prediction model, and the fitness curve is shown in Fig. 5.

**Figure 6:**
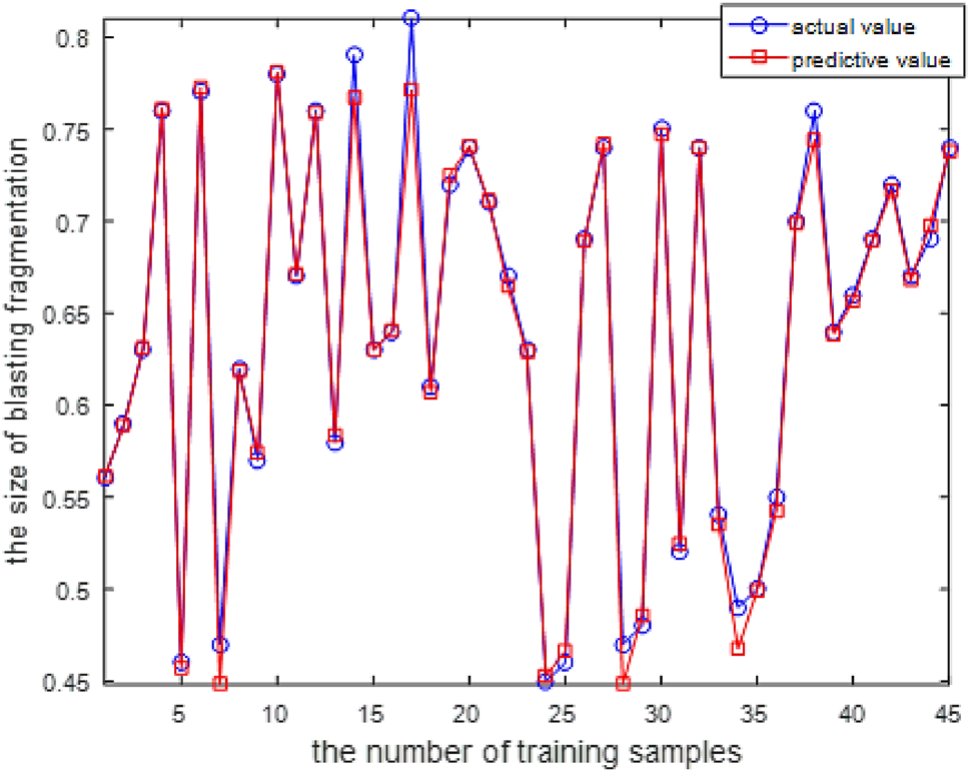
Blasting fragmentation training effect chart of KPCA-BAS-BP model.

Figure 5 shows that the fitness value of the sample reaches a stable state after 12 iterations, and the MSE of the prediction model is $9.9428\times {10}^{-4}$. The optimal initial weight and threshold value obtained at this time are substituted into the network model to simulate the training sample. The training effect is shown in Fig. 6.

### Comparative analysis with other models

**Figure 7:**
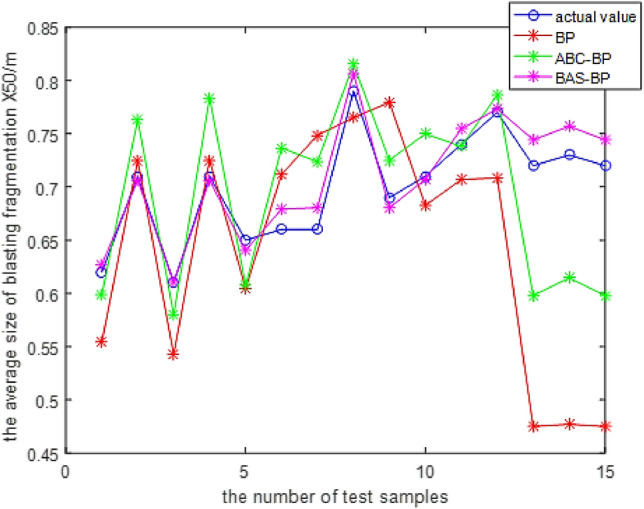
Test results of different prediction models.

The training effect proves that the model has good learning ability. To verify that the KPCA-BAS-BP model also has excellent generalization ability, 15 sets of sample data are used for testing, and they are compared with the unoptimized BP neural network model and the ABC-BP neural network model (Fig. 7).

Tables 2 and 3 show that the prediction effect of BP neural network is poor, with the mean relative error(MRE) of 12.62% and the root mean square error(RMSE) of 12.12%. The optimal solution is obtained in 35 iterations. Meanwhile, the MRE of the ABC-BP model is 8.01%, and the RMSE is 6.74%. The optimal solution is obtained in 22 iterations, and the accuracy of the BP neural network optimized by the ABC algorithm is improved. As regards the KPCA-BAS-BP model, the MRE is 1.77%, which is 10.85% (12.62–1.77%) and 6.24% (8.01–1.77%) lower than unoptimized BP and ABC-BP, respectively, the RMSE is 1.52%, which is 10.6% (12.12–1.52%) and 5.22% (6.74–1.52%) lower than unoptimized BP neural network and ABC-BP, respectively. The optimal solution is obtained in 12 iterations, which is lower than those of the BP neural network (23 (35–12) iterations) and the ABC-BP (10 (22–12) iterations) iterative optimization. These findings show that the BP neural network prediction model based on KPCA and BAS algorithm has higher prediction accuracy and convergence rate and can accurately predict blasting fragmentation.

**Table 2:** Comparison of relative errors of three prediction models.

Output variable	actual value	KPCA-BAS-BP model		ABC-BP model		BP neural network	
		predictive value	relative error	predictive value	relative error	Predictive value	relative error
The average size of blasting fragmentation X50/m	0.62	0.6272	0.0117	0.5986	0.0346	0.5540	0.1065
	0.71	0.7062	0.0053	0.7635	0.0754	0.7250	0.0211
	0.61	0.6106	0.0009	0.5797	0.0497	0.5424	0.1108
	0.71	0.7062	0.0053	0.7835	0.1035	0.7250	0.0211
	0.65	0.6402	0.0150	0.6076	0.0652	0.6047	0.0697
	0.66	0.6791	0.0289	0.7366	0.1161	0.7118	0.0784
	0.66	0.6805	0.0311	0.7236	0.0964	0.7485	0.1341
	0.79	0.8068	0.0212	0.8154	0.0321	0.7652	0.0314
	0.69	0.6804	0.0139	0.7252	0.0509	0.7795	0.1297
	0.71	0.7072	0.0040	0.7499	0.0562	0.6827	0.0385
	0.74	0.7544	0.0195	0.7381	0.0026	0.7063	0.0447
	0.77	0.7733	0.0043	0.7866	0.0215	0.7085	0.0799
	0.72	0.7443	0.0338	0.5980	0.1694	0.4751	0.3401
	0.73	0.7569	0.0369	0.6145	0.1583	0.4769	0.3467
	0.72	0.7444	0.0339	0.5980	0.1694	0.4750	0.3403

**Table 3:** Comparison of evaluation indexes of the three models.

Predictive model	MRE (%)	RMSE (%)	Number of iterations for optimal solution
BP	12.62	12.12	35
ABC-BP	8.01	6.74	22
KPCA-BAS-BP	1.77	1.52	12

## Conclusions

1. Propose a new beetle search algorithm to improve the BP neural network model, which compensates for the shortcomings of long training time, overfitting, and high model training complexity of a single BP neural network.
2. The kernel principal component analysis method was used to reduce the dimensionality of the input sample data, effectively reducing information duplication and interference, improving the accuracy of the prediction model, and greatly improving the training speed.
3. Selecting 60 sets of measured data from the Beskuduk open-pit coal mine for modeling, the average relative error of the model is 1.77%, and the mean square error is 1.52%. Compared with other prediction models, the KPCA-BAS-BP model has the highest prediction accuracy, providing a new approach for predicting the blasting vibration block size of open-pit coal mines in complex environments.

## Data Availability

The datasets used and analysed during the current study available from the corresponding author on reasonable request.
